# A Prediction-Based Spatial-Spectral Adaptive Hyperspectral Compressive Sensing Algorithm

**DOI:** 10.3390/s18103289

**Published:** 2018-09-30

**Authors:** Ping Xu, Bingqiang Chen, Lingyun Xue, Jingcheng Zhang, Lei Zhu

**Affiliations:** College of Life Information & Instrument Engineering, Hangzhou Dianzi University, Hangzhou 310018, China; xuping@hdu.edu.cn (P.X.); cbq@hdu.edu.cn (B.C.); zhulei@hdu.edu.cn (L.Z.)

**Keywords:** hyperspectral images, compressive sensing, spatial-spectral adaptation, interspectral prediction

## Abstract

In order to improve the performance of storage and transmission of massive hyperspectral data, a prediction-based spatial-spectral adaptive hyperspectral compressive sensing (PSSAHCS) algorithm is proposed. Firstly, the spatial block size of hyperspectral images is adaptively obtained according to the spatial self-correlation coefficient. Secondly, a k-means clustering algorithm is used to group the hyperspectral images. Thirdly, we use a local means and local standard deviations (LMLSD) algorithm to find the optimal image in the group as the key band, and the non-key bands in the group can be smoothed by linear prediction. Fourthly, the random Gaussian measurement matrix is used as the sampling matrix, and the discrete cosine transform (DCT) matrix serves as the sparse basis. Finally, the stagewise orthogonal matching pursuit (StOMP) is used to reconstruct the hyperspectral images. The experimental results show that the proposed PSSAHCS algorithm can achieve better evaluation results—the subjective evaluation, the peak signal-to-noise ratio, and the spatial autocorrelation coefficient in the spatial domain, and spectral curve comparison and correlation between spectra-reconstructed performance in the spectral domain—than those of single spectral compression sensing (SSCS), block hyperspectral compressive sensing (BHCS), and adaptive grouping distributed compressive sensing (AGDCS). PSSAHCS can not only compress and reconstruct hyperspectral images effectively, but also has strong denoise performance.

## 1. Introduction

Hyperspectral images contain both the spatial and spectral characteristics. In recent years, they have been widely used in agriculture and forestry research, marine monitoring, natural disaster monitoring, and military reconnaissance [1]. However, with the increasing development of remote sensing technology, the requirement to increase the resolution of hyperspectral data has led to an extreme increase in its amount, which has caused tremendous pressure on the transmission and storage of hyperspectral images [2,3]. Solving this problem can start from the hardware itself, such as increasing the storage space of the hardware. However, attempting to solve this problem from the hardware will inevitably raise the cost significantly, and finally turn the problem into an expensive hardware cost problem. Another feasible means to solve this problem is to perform effective data compression and solve the problem at the data source in the form of a small amount of information to represent all the information.

The compressed sensing theory was proposed by Donoho et al. in 2006 [4]. The theory states that if the signal is sparse itself or in a certain transform domain, the signal can be sampled with much less data than those of Nyquist sampling criterion, and reconstructed accurately with these sampled data [5]. Berger [6] pointed out that the high correlation of the signal itself will help improve the compression ratio and the reconstructed quality of compressed sensing. Unlike ordinary 2D images, hyperspectral images contain high interspectral and interspatial correlation. How to make full use of these characteristics of hyperspectral images to improve reconstruction performance is a hot research field of hyperspectral compressive sensing. Huang et al. proposed a block compressive sensing (BCS) of hyperspectral images based on prediction error [7]. Lin et al. proposed a hyperspectral image compression algorithm based on adaptive band grouping [8]. Zhang proposed a structured sparsity-based hyperspectral blind compressive sensing (SSHBCS) method to sparsify hyperspectral images [9]. Spatial autocorrelation coefficients were involved in the strategy of spatial adaptive partitioning to determine the size of the block [10]. Gao pointed out that the k-means clustering algorithm was suitable for spectral adaptive grouping [11]. Gaussian measurement matrix [12], the discrete cosine transform (DCT) sparse dictionary [13,14], and the stagewise orthogonal matching pursuit (StOMP) algorithm [15,16] were used in the hyperspectral compressive sensing. Xu et al. proposed an adaptive grouping distributed compressive sensing reconstruction (AGDCS) of plant hyperspectral data [17]. A sparse and low-rank near-isometric linear embedding (SLRNILE) method based on the John-Lindenstrauss lemma for dimensionality reduction and to extract proper features for hyperspectral imagery (HSI) classification [18]. A robust kernel archetypoid analysis (RKADA) method was proposed to extract pure endmembers from HSI [19], in which each pixel is assumed to be a sparse linear mixture of all endmembers and each endmember corresponds to a real pixel in the image scene. A fast and robust principal component analysis on Laplacian graph (FRPCALG) method was proposed to select bands of hyperspectral imagery [20].

In our previous research [21], we have developed SSCS technology for plant hyperspectral data in the spectral domain. Huang et al. introduced BCS for hyperspectral images in the spatial domain [7]. Hyperspectral images have strong spectral and spatial correlations. The compressive sensing of hyperspectral images using both the spectral and spatial correlations can further improve their sparse representation, which is also able to improve the accuracy of reconstruction. Therefore, the strategies of interspatial blocking and interspectral grouping are still needed to be further studied. In order to further improve the compression and reconstruction performance of hyperspectral compressive sensing, adaptively interspatial blocking strategy, adaptively interspectral grouping strategy and linear interspectral prediction technology are integrated to construct the new prediction-based spatial-spectral adaptive hyperspectral compressive sensing (PSSAHCS) algorithm, which can not only compress and reconstruct hyperspectral images effectively, but also have strong denoising performance.

In this paper, the row correlation and column correlation of hyperspectral images are studied according to the spatial autocorrelation coefficients [22], and used to determine the optimal block size. In addition, after analyzing the interspectral correlation of adjacent bands [22,23,24,25], the introduction of a k-means clustering algorithm [26,27,28] is used to group the hyperspectral images in the spectral domain, and all highly correlated bands are divided into the same group. At the same time, it can be seen that the correlation of some adjacent bands decreases significantly according to the spectral correlation curve, and that the spectral curves are very jittery near these bands. Gao [11] pointed out that this phenomenon is caused by the significant absorption of electromagnetic waves in these bands by the atmosphere, which means that the images in these bands contain a lot of noise. Therefore, this paper introduced the idea of intragroup prediction to improve the reconstruction quality of these noise bands. The reference image is chosen in the group, and then the rest of the images in the group are predicted using the reference image. The residual image can be calculated by using the intragroup reference image to subtract the intragroup prediction image, and then the residual image is encoded and compressed. Additionally, the residual image is reconstructed using a reconstruction algorithm. Finally, the reconstructed image can be obtained by the reconstructed residual image and the reference image [29].

## 2. Methods

### 2.1. PSSAHCS Algorithm

**Figure 1:**
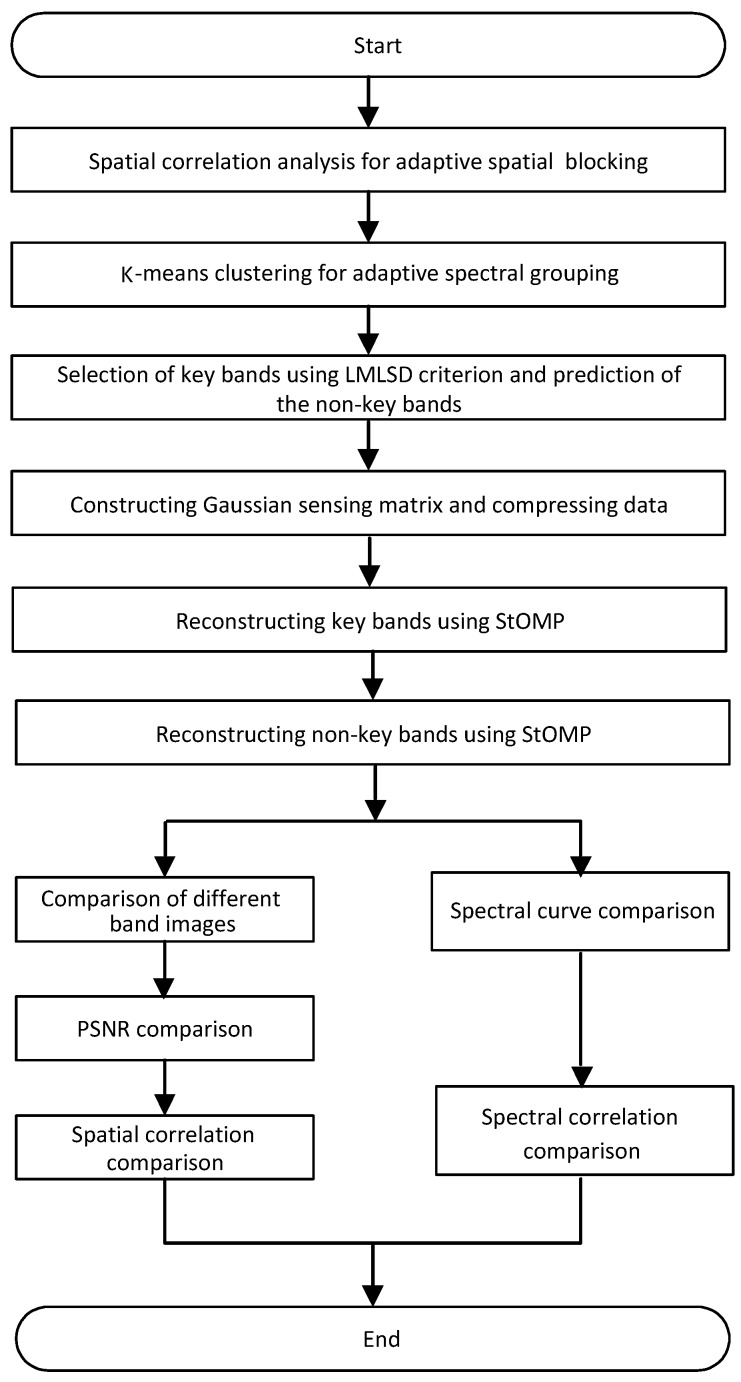
The flowchart of the prediction-based spatial-spectral adaptive hyperspectral compressive sensing (PSSAHCS) algorithm and experiments.

Figure 1 is the flowchart of the PSSAHCS algorithm and experiments. Firstly, the spatial correlation of hyperspectral images is analyzed and the appropriate ranges of row correlation coefficients and column correlation coefficients are obtained to determine the spatial block size. Secondly, the spectral correlation of the adjacent bands is calculated in the spectral domain and the grouping of hyperspectral images is adaptively decided using the k-means clustering algorithm. Thirdly, the local means and local standard deviations (LMLSD) criterion is used to choose the optimal band with the lowest noise as the key band in a group, and the non-keys bands are linearly predicted according to the key bands. Fourthly, the Gaussian measurement matrix is used to compress key bands, and DCT is used as the sparse dictionary combining with Gaussian measurement to structure the sensor matrix. Finally, the reconstruction results are evaluated from the spatial domain and the spectral domain, respectively. The spatial evaluation is performed from the three perspectives of the subjective evaluation, the peak signal-to-noise ratio, and the spatial autocorrelation coefficient. The spectral evaluation is performed using two levels: spectral curve comparison and correlation between spectra.

### 2.2. Adaptive Spatial Blocking

Similar to ordinary two-dimensional images, hyperspectral images show a certain spatial correlation. The spatial correlation of hyperspectral images is caused by the similarities between the local structures of the objects, adjacent pixels or similar pixels in the same band. The spatial correlation is generally expressed by the spatial correlation coefficient, η(Δ*x*, Δ*y*), as follows:

(1) $$\eta \left(\Delta x,\Delta y,z\right)=\iint f_{x,y,z}\times f_{x+\Delta x,y+\Delta y,z}dxdy$$

where $f_{x,y,z}$ represents the gray value of the *x*-th row and the *y*-th column pixel in the *z*-th band. Δ*x*, Δ*y* represent the distances between the target pixel and the current pixel, respectively. Because the above equation is not convenient for calculation, it is discretized and normalized to the following equation:

(2) $$\eta \left(\Delta x,\Delta y,z\right)=\frac{\sum_{x=1}^{a}\sum_{y=1}^{b}\left(f_{x,y,z}-\bar{f_{z}}\right)\times \left(f_{x+\Delta x,y+\Delta y,z}-\bar{f_{z}}\right)}{\sum_{x=1}^{a}\sum_{y=1}^{b}{\left(f_{x,y,z}-\bar{f_{z}}\right)}^{2}}$$

where *a* and *b* represent the number of rows and columns of the image, respectively; $\bar{f_{z}}$ denotes the average gray level of the *z*-th band image of the hyperspectral image.

(3) M=min(Δx,Δy,z) s.t. 0.9≤η(Δx,Δy,z)≤0.95

where M is the block size.

### 2.3. Adaptive Spectral Grouping

#### 2.3.1. Adaptive Spectral Grouping Using k-Means Clustering Algorithm

For the distribution of spectral correlation, high-correlation bands should be divided into the same group to make full use of the interspectral redundancy. The k-means clustering algorithm is used to group camellia sinensishyperspectral images.

The basic idea of the k-means clustering algorithm is as follows: In the initial stage, it is necessary to give k centroids as the initial k cluster centers, and then calculate the distance between each sample and k centroids. Each class recalculates the mean value as the new k centroids. Finally, repeat the above steps until the centroids do not change.

In the k-means clustering algorithm, the Euclidean distance is generally used to measure the distance between the samples and the centroid. For tea hyperspectral images, the distance between each sample and the centroid can be calculated as follows:

(4) $$D\left(z_{i},z_{c}\right)=\sqrt{\sum_{x=1}^{a}\sum_{y=1}^{b}{\left(f_{x,y,z_{i}}-f_{x,y,z_{c}}\right)}^{2}}$$

where $z_{i}$ denotes the band, and $z_{c}$ denotes thezc cluster centroid.

#### 2.3.2. LMLSD

After the spectral clustering is grouped, it is necessary to select the image with the least noise from the group as the key image. In this paper, LMLSD [18] is used to find the minimum noise image in the group. The calculation equations of LMLSD are as follows:

(5) $$M_{num}\left(z\right)=\frac{1}{a\times b}\sum_{i=1}^{a}\sum_{j=1}^{b}f\left(i,j,z\right)$$

(6) $$D_{num}\left(z\right)=\sqrt{\frac{1}{\left(a\times b-1\right)}\sum_{i=1}^{a}\sum_{j=1}^{b}{\left(f_{num}\left(i,j,z\right)-M_{num}(z)\right)}^{2}}$$

(7) $$R\left(z\right)=20lg\frac{M_{mean}\left(z\right)}{D_{mean}\left(z\right)}$$

In Equation (5), *a*, *b* are the row and column of the sub-block image, respectively, *z* is the *z*-th band in the group, *f_num_* is the *num*-th sub-block, and *M_num_* is the mean gray value of the *num*-th sub-block. In Equation (6), *D_num_* is the standard deviation of the *num*-th sub-block. After obtaining the maximum and minimum values of *D_num_*, we can get the count of sub-blocks in the same interval, and calculate the mean gray *M_mean_* and standard deviation *D_mean_* of the all sub-blocks in the interval with the most sub-blocks. In Equation (7), *R* is the PSNR value of LMLSD.

#### 2.3.3. Spectral Grouping Based on Linear Prediction

After grouping by the k-means clustering algorithm, there is a high interspectral correlation in each group, so the linear predictor can be used to predict the images in the group. The linear prediction model is shown,

(8) $$f_{g}\left(x,y\right)=m\times f_{R}\left(x,y\right)+n$$

where $f_{R}\left(x,y\right)$ is the gray value of the pixel of the *x*-th row and the *y*-th column of the reference image in the group; $f_{g}\left(x,y\right)$ is the gray value of the pixel of the *x*-th row and *y*-th column of the image to be predicted in the group; and *m* and *n* are prediction coefficients. 

Assuming that the size of the image is a×b, the prediction error of each image to be predicted can be

(9) $$\varepsilon =\sqrt{\frac{1}{a\times b}\sum_{x=1}^{a}\sum_{y=1}^{b}{\left(f_{g}\left(x,y\right)-m\times f_{R}\left(x,y\right)-n\right)}^{2}}$$

For Equation (9), in order to minimize *ε*, we need to satisfy Equations (10) and (11):

(10) $$\frac{\partial \varepsilon^{2}}{\partial m}=0$$

(11) $$\frac{\partial \varepsilon^{2}}{\partial n}=0$$

According to Equations (9)–(11), the solutions for *m* and *n* can be obtained respectively, as shown in Equations (12) and (13):

(12) $$m=\frac{R\left(f_{R}\left(x,y\right),f_{g}\left(x,y\right)\right)-u\left(f_{R}\left(x,y\right)\right)\times u\left(f_{g}\left(x,y\right)\right)}{R\left(f_{R}\left(x,y\right),f_{R}\left(x,y\right)\right)-u{\left(f_{R}\left(x,y\right)\right)}^{2}}$$

(13) $$n=u\left(f_{g}\left(x,y\right)\right)-\frac{R\left(f_{R}\left(x,y\right),f_{g}\left(x,y\right)\right)-u\left(f_{R}\left(x,y\right)\right)\times u\left(f_{g}\left(x,y\right)\right)}{R\left(f_{R}\left(x,y\right),f_{R}\left(x,y\right)\right)-u{\left(f_{R}\left(x,y\right)\right)}^{2}}\times u\left(f_{R}\left(x,y\right)\right)$$

where

(14) $$R\left(f_{R}\left(x,y\right),f_{g}\left(x,y\right)\right)=\frac{1}{a\times b}\sum_{x=1}^{a}\sum_{y=1}^{b}f_{R}\left(x,y\right)\times f_{g}\left(x,y\right)$$

(15) $$u\left(f_{R}\left(x,y\right)\right)=\frac{1}{a\times b}\sum_{x=1}^{a}\sum_{y=1}^{b}f_{R}\left(x,y\right)$$

(16) $$u\left(f_{g}\left(x,y\right)\right)=\frac{1}{a\times b}\sum_{x=1}^{a}\sum_{y=1}^{b}f_{g}\left(x,y\right)$$

After the prediction is completed, the prediction residual for a certain pixel is obtained by subtracting the predicted value from the actual gray value of the pixel. The residual image of the prediction is compressed, and the reconstructed image is then added to the corresponding predicted image to obtain the reconstructed image.

### 2.4. Stagewise Orthogonal Matching Pursuit Algorithm

The stagewise orthogonal matching pursuit (StOMP) algorithm was proposed by Donoho et al. in 2012 [30]. The algorithm is an improved algorithm of orthogonal matching pursuit (OMP) [31]. Compared with the OMP algorithm, this algorithm selects multiple atoms per iteration. Therefore, the number of iterations is lower than that of the OMP algorithm, which greatly improves the reconstruction efficiency while ensuring the reconstruction accuracy.

### 2.5. The Evaluation Measures

#### 2.5.1. PSNR

Peak signal-to-noise ratio (PSNR) is chosen to evaluate the reconstructed performance in the spatial domain; mean square error (MSE) and PSNR are defined by

(17) $$\mathrm{MSE}=\frac{1}{a\times b\times c}\sum_{x=1}^{a}\sum_{y=1}^{b}\sum_{z=1}^{c}{\left|f_{ori}\left(x,y,z\right)-f_{rec}\left(x,y,z\right)\right|}^{2}$$

where *a*, *b* and *c* are the row, column and band count of the hyperspectral images, respectively, $f_{ori}$ is the original image, and $f_{rec}$ is the reconstructed image.

(18) $$\mathrm{PSNR}=10\times log_{10}\frac{{\left(2^{n}-1\right)}^{2}}{MSE}$$

where *n* is the bits of the image.

#### 2.5.2. Interspectral Correlation

The interspectral correlation of hyperspectral images is formed by the reflection of a certain object in different wavebands, and there is a high correlation between adjacent pixels at the same spatial position in different bands.

The interspectral correlation in hyperspectral images is usually expressed by the spectral correlation coefficient $\zeta \left(z_{1},z_{2}\right)$. The calculation of the spectral correlation coefficient is shown in Equation (19):

(19) $$\zeta \left(z_{1},z_{2}\right)=\frac{\sum_{x=1}^{a}\sum_{y=1}^{b}\left(f_{x,y,z_{1}-{\bar{f}}_{z_{1}}}\right)\times \left(f_{x,y,z_{2}-{\bar{f}}_{z_{2}}}\right)}{\sqrt{\sum_{x=1}^{a}\sum_{y=1}^{b}{\left(f_{x,y,z_{1}-{\bar{f}}_{z_{1}}}\right)}^{2}\times \sum_{x=1}^{a}\sum_{y=1}^{b}{\left(f_{x,y,z_{2}-{\bar{f}}_{z_{2}}}\right)}^{2}}}$$

where $z_{1}$ and *z*_2_ represent different bands of hyperspectral images, respectively.

## 3. Experimental Results and Discussion

### 3.1. Data Description

A visible and near-infrared hyperspectral imaging system covering the spectral wavelengths of 380–1030 nm was used in this study. The system includes an imaging spectrograph, a charge coupled device (CCD) camera (C8484-05, Hamamatsu City, Japan), a lens, two light sources provided by two 150 W quartz tungsten halogen lamps and V10E software (Isuzu Optics Corp, Hsinchu County, Taiwan) for operating the hyperspectral image system. The area CCD array detector of the camera has 6726512 pixels and the spectral resolution is 2.8 nm. The data used in the experiment are hyperspectral images of 12 pieces of camellia sinensis. A single pixel is defined by a 12-bit unsigned integer and the resolution of the processed image is 128 × 256. 

### 3.2. Performance Evaluation in the Spatial Domain

#### 3.2.1. Subjective Performance Comparison

**Figure 2:**
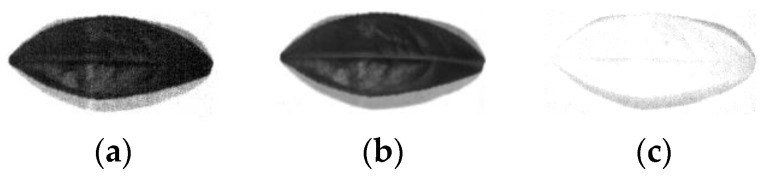
Original images of different wavelengths. (**a**) 440 nm; (**b**) 660 nm; (**c**) 980 nm.

**Figure 3:**
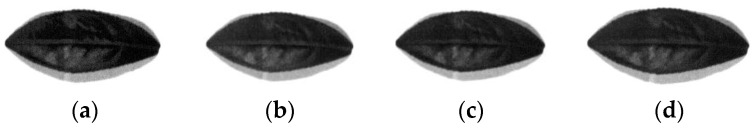
Reconstructed images of the 440 nm band of single spectral compression sensing (SSCS). (**a**) 0.10 bytes per pixel (bpp); (**b**) 0.15 bpp; (**c**) 0.20 bpp; (**d**) 0.25 bpp.

**Figure 4:**
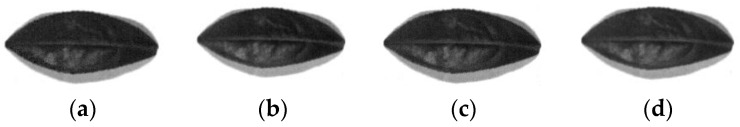
Reconstructed images of the 660 nm band of SSCS. (**a**) 0.10 bpp; (**b**) 0.15 bpp; (**c**) 0.20 bpp; (**d**) 0.25 bpp.

**Figure 5:**
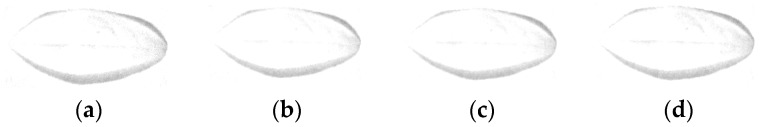
Reconstructed images of the 980 nm band of SSCS. (**a**) 0.10 bpp; (**b**) 0.15 bpp; (**c**) 0.20 bpp; (**d**) 0.25 bpp.

**Figure 6:**
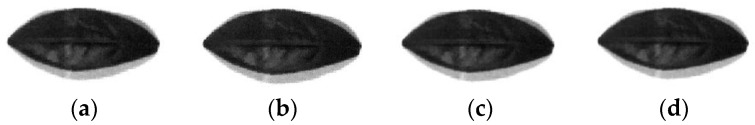
Reconstructed images of the 440 nm band of BHCS. (**a**) 0.10 bpp; (**b**) 0.15 bpp; (**c**) 0.20 bpp; (**d**) 0.25 bpp.

**Figure 7:**
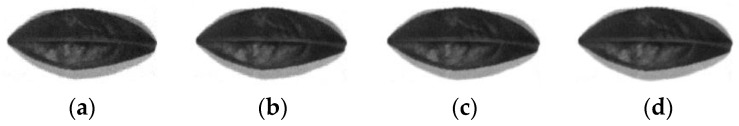
Reconstructed images of the 660 nm band of BHCS. (**a**) 0.10 bpp; (**b**) 0.15 bpp; (**c**) 0.20 bpp; (**d**) 0.25 bpp.

**Figure 8:**
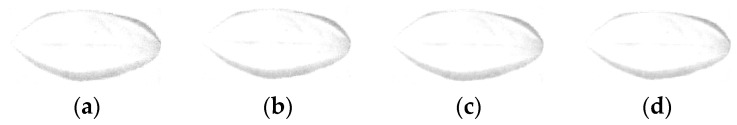
Reconstructed images of the 980 nm band of BHCS. (**a**) 0.10 bpp; (**b**) 0.15 bpp; (**c**) 0.20 bpp; (**d**) 0.25 bpp.

**Figure 9:**
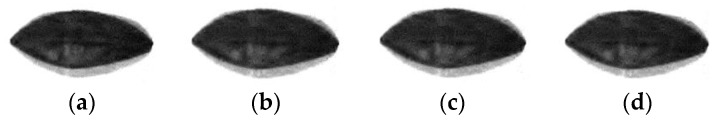
Reconstructed images of the 440 nm band of AGDCS. (**a**) 0.10 bpp; (**b**) 0.15 bpp; (**c**) 0.20 bpp; (**d**) 0.25 bpp.

**Figure 10:**
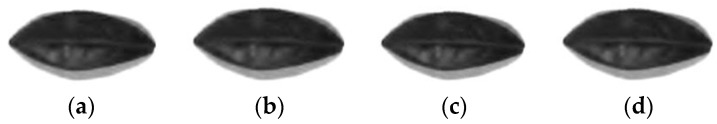
Reconstructed images of the 660 nm band of AGDCS. (**a**) 0.10 bpp; (**b**) 0.15 bpp; (**c**) 0.20 bpp; (**d**) 0.25 bpp.

**Figure 11:**
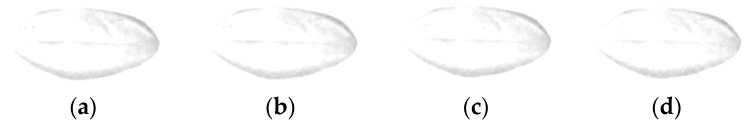
Reconstructed images of the 980 nm band of AGDCS. (**a**) 0.10 bpp; (**b**) 0.15 bpp; (**c**) 0.20 bpp; (**d**) 0.25 bpp.

**Figure 12:**
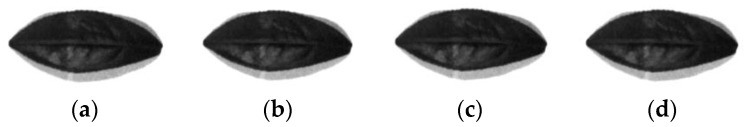
Reconstructed images of the 440 nm band of PSSAHCS. (**a**) 0.10 bpp; (**b**) 0.15 bpp; (**c**) 0.20 bpp; (**d**) 0.25 bpp.

**Figure 13:**
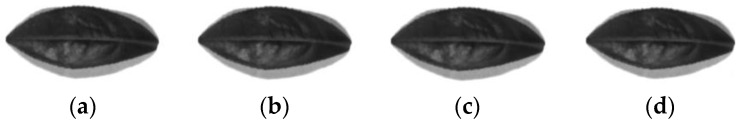
Reconstructed images of the 660 nm band of PSSAHCS. (**a**) 0.10 bpp; (**b**) 0.15 bpp; (**c**) 0.20 bpp; (**d**) 0.25 bpp.

**Figure 14:**
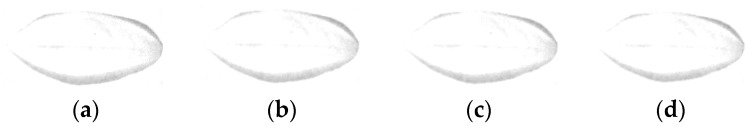
Reconstructed images of the 980 nm band of PSSAHCS. (**a**) 0.10 bpp; (**b**) 0.15 bpp; (**c**) 0.20 bpp; (**d**) 0.25 bpp.

SSCS, block hyperspectral compressive sensing (BHCS), AGDCS and PSSAHCS are also used to give the experimental results. Figure 2 shows the original images of the 440 nm, 620 nm and 980 nm bands. Figure 3, Figure 4, Figure 5, Figure 6, Figure 7, Figure 8, Figure 9, Figure 10, Figure 11, Figure 12, Figure 13 and Figure 14 show the reconstructed hyperspectral images of the 440 nm, 620 nm and 980 nm bands at different bit rates for different algorithms. It can be seen from Figure 3, Figure 4, Figure 5, Figure 6, Figure 7, Figure 8, Figure 9, Figure 10, Figure 11, Figure 12, Figure 13 and Figure 14 that the subject quality of the reconstructed hyperspectral images at different bit rates become better, especially for the details such as edges, veins, leaf stems and so on, when the bit rate rises for all algorithms. At the same time, it can be seen that for the 620 nm image with no significant noise, BHCS and PSSAHCS can achieve a good reconstruction effect for different bit rates. For the reconstructed 440 nm and 980 nm images with significant noise, there is “edge effect” for SSCS, BHCS and AGDCS at low bit rates, while PSSAHCS can denoise effectively. Therefore, PSSAHCS can not only retain the details of the original image, but also remove the noise effectively at different bit rates.

#### 3.2.2. The Peak Signal-to-Noise Ratio (PSNR) Performance Comparison

**Figure 15:**
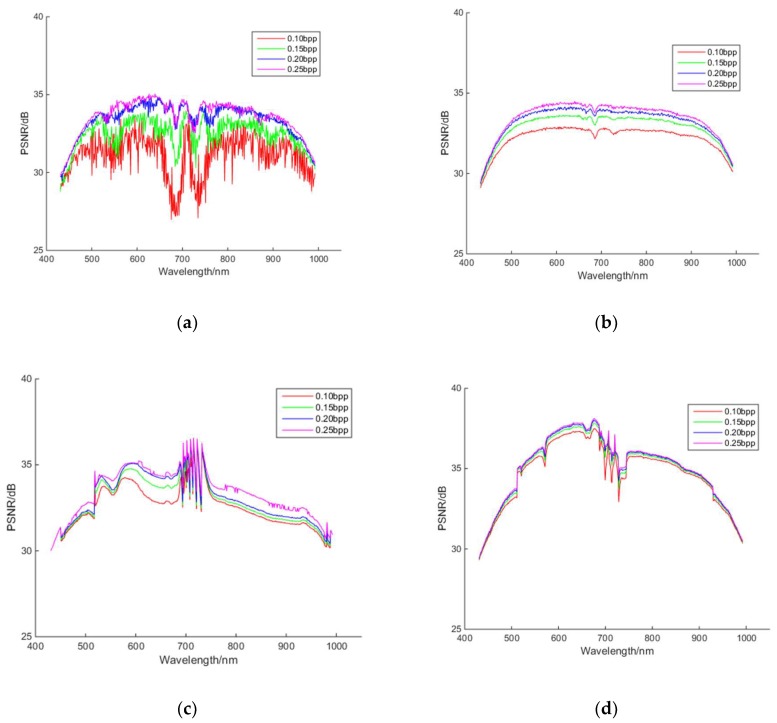
PSNR of reconstructed images at different bit rates for different algorithms. (**a**) SSCS; (**b**) BHCS; (**c**) AGDCS; (**d**) PSSAHCS.

Figure 15 shows the PSNR of the reconstructed hyperspectral images of the 440 nm, 620 nm and 980 nm bands at different bit rates for different algorithms. As it can be seen from Figure 15, with the increase of the bit rate, the fidelity of reconstructed images of all algorithms can be improved. The reconstructed PSNRs of PSSAHCS for most bands are significantly higher than those of SSCS and BHCS at different bit rates. Table 1 shows that the average PSNR of most bands of PSSAHCS are significantly higher than those of SSCS, BHCS and AGDCS at the same compression rates. 

Table 1 shows the average PSNR for the different algorithms at different bit rates. PSSAHCS can achieve about 2 dB higher average PSNR than that of SSCS, BHCS and AGDCS.

#### 3.2.3. Comparison of Spatial Correlation

**Figure 16:**
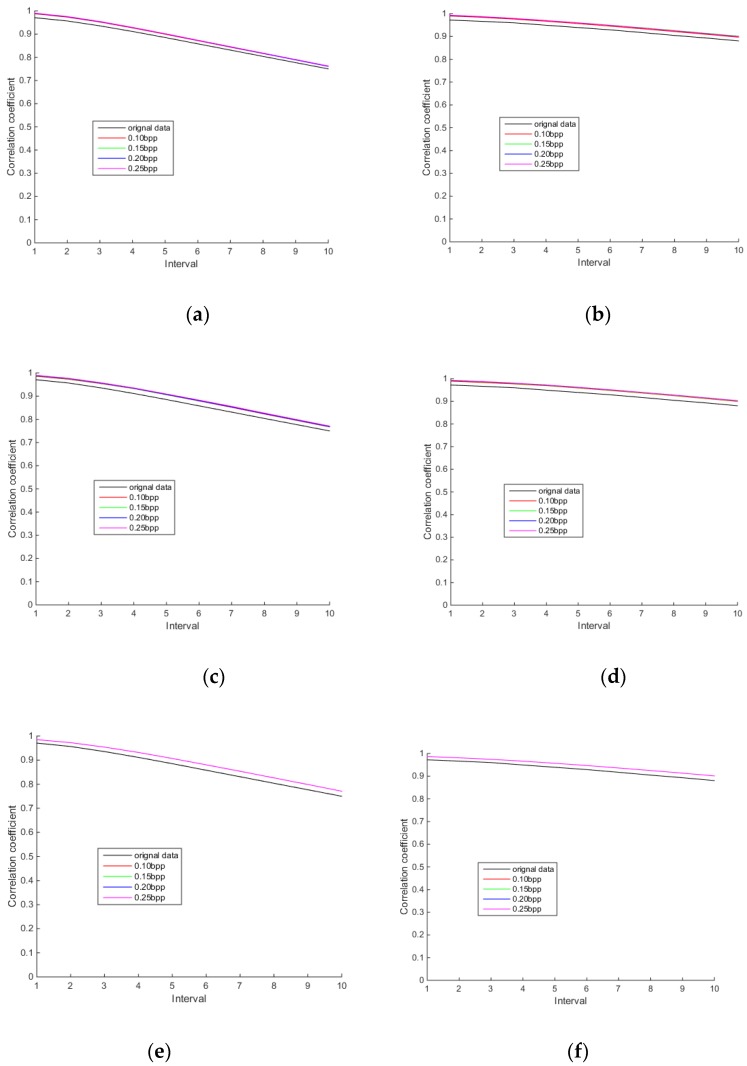

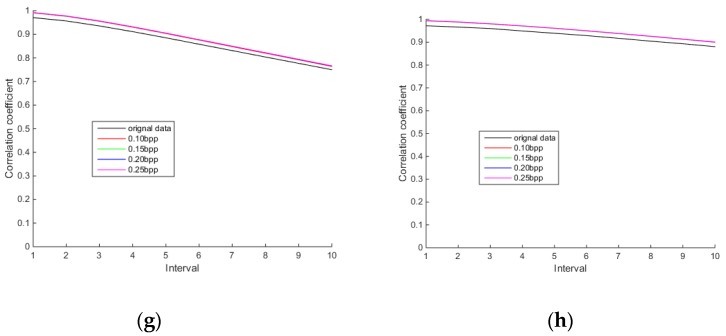
Row and column correlations for the 440 nm band of different algorithms. (**a**) Row correlation of SSCS; (**b**) column correlation of SSCS; (**c**) row correlation of BHCS; (**d**) column correlation of BHCS; (**e**) row correlation of AGDCS; (**f**) column correlation of AGDCS; (**g**) row correlation of PSSAHCS; (**h**) column correlation of PSSAHCS.

**Figure 17:**
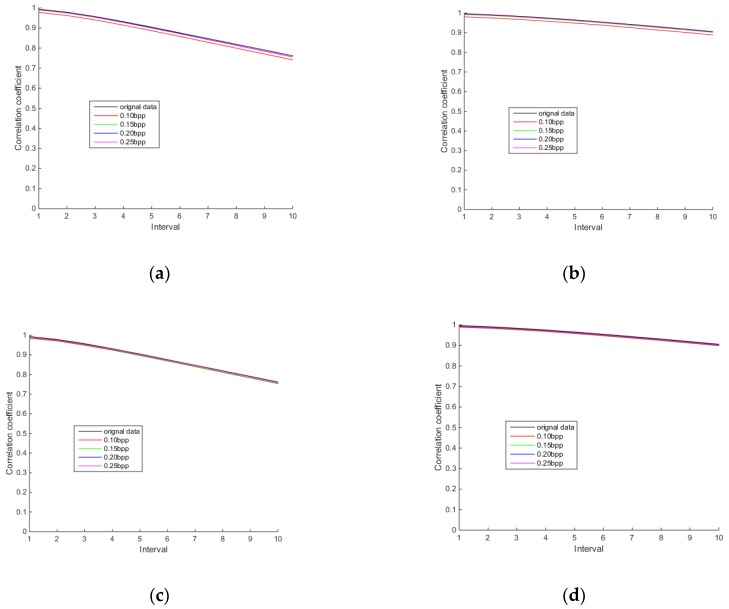

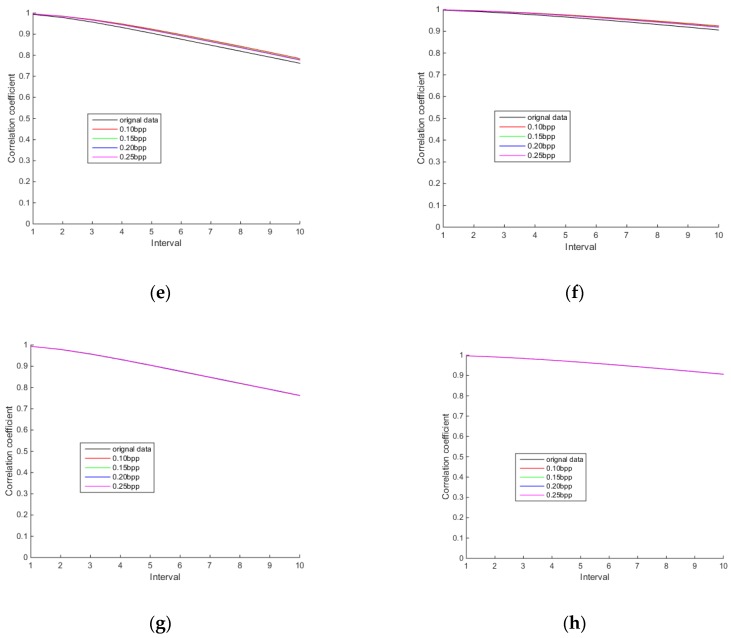
Row and column correlations for the 620 nm band of different algorithms. (**a**) Row correlation of SSCS; (**b**) column correlation of SSCS; (**c**) row correlation of BHCS; (**d**) column correlation of BHCS; (**e**) row correlation of AGDCS; (**f**) column correlation of AGDCS; (**g**) row correlation of PSSAHCS; (**h**) column correlation of PSSAHCS.

**Figure 18:**
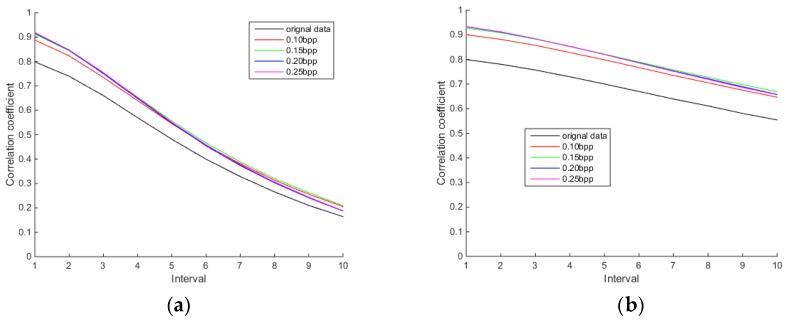

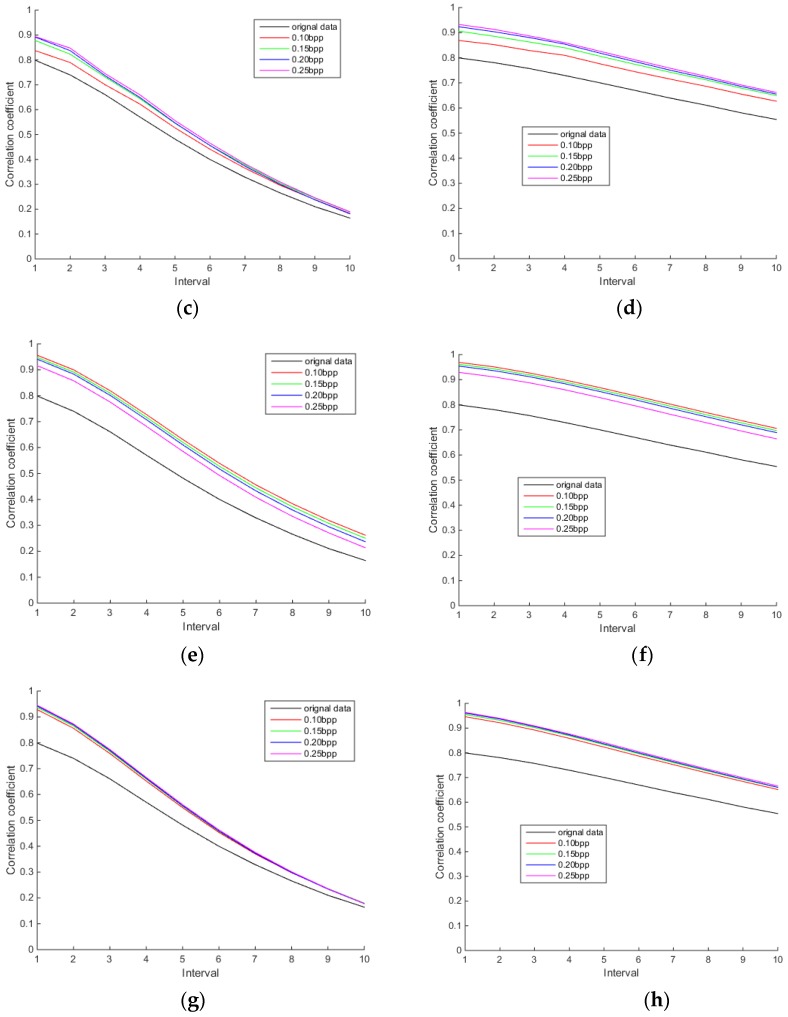
Row and column correlations for the 980 nm band of different algorithms. (**a**) Row correlation of SSCS; (**b**) column correlation of SSCS; (**c**) row correlation of BHCS; (**d**) column correlation of BHCS; (**e**) row correlation of AGDCS; (**f**) column correlation of AGDCS; (**g**) row correlation of PSSAHCS; (**h**) column correlation of PSSAHCS.

Spatial correlation is one of the characteristics of hyperspectral images. Figure 16, Figure 17 and Figure 18 show the row and column correlation curves of the reconstructed hyperspectral images of 440 nm, 620 nm and 980 nm of different algorithms at different bit rates. It can be seen that the row correlation and column correlation curves of reconstructed tea hyperspectral images and the original images show the same trend, that is, as the interval increases, the correlation drops. In addition, the row correlations and column correlations of different reconstructed algorithms at 440 nm and 980 nm are higher than the row correlations and column correlations of the original image. This is because the StOMP reconstruction algorithm has a certain denoising ability, and the correlation is obviously improved after denoising. Moreover, it also shows that the row correlation and column correlation of PSSAHCS is slightly higher than that of SSCS, BHCS and AGDCS for different bands. 

### 3.3. Comparison in the Spectral Domain

#### 3.3.1. Comparison of Spectral Curve

**Figure 19:**
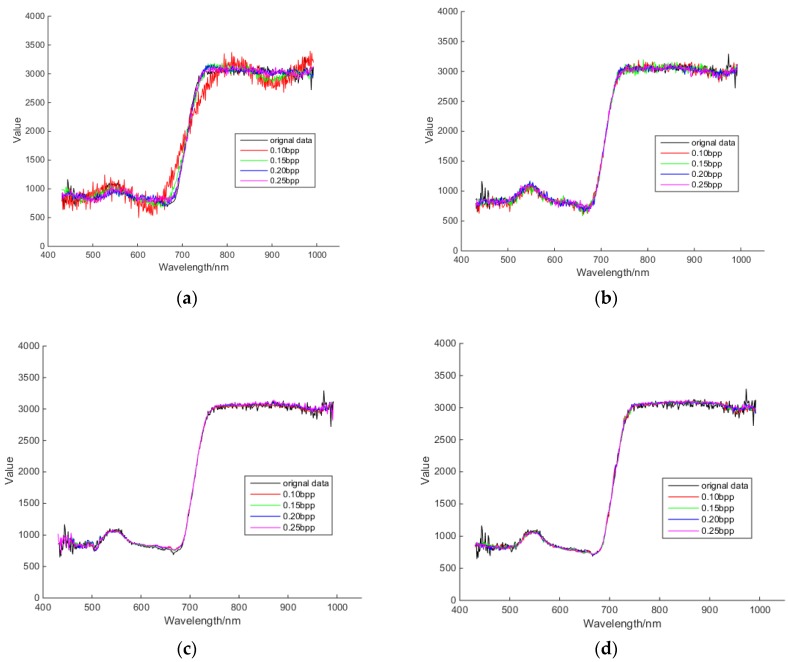
Spectral curve comparison of different algorithms at different bit rates. (**a**) SSCS; (**b**) BHCS; (**c**) AGDCS; (**d**) PSSAHCS.

A spectral curve is an important way to describe and distinguish different features in hyperspectral images. Figure 19 shows the reconstructed spectral curves of different algorithms at different compression rates. It can be seen that the reconstructed spectral curves of different algorithms are closer and closer to the original spectral curves as the compression rate increases. PSSAHCS puts similar bands into the same group, and then uses the prediction algorithm to perform linear prediction to improve the degree of linearity within the group. Therefore, the reconstructed spectral curves of PSSAHCS are obviously smoother than those of SSCS, BHCS and AGDCS at different bit rates. At the same time, the linear prediction algorithm plays a role in removing noise and is useful for hyperspectral imagery.

#### 3.3.2 Spectral Correlation Comparison

**Figure 20:**
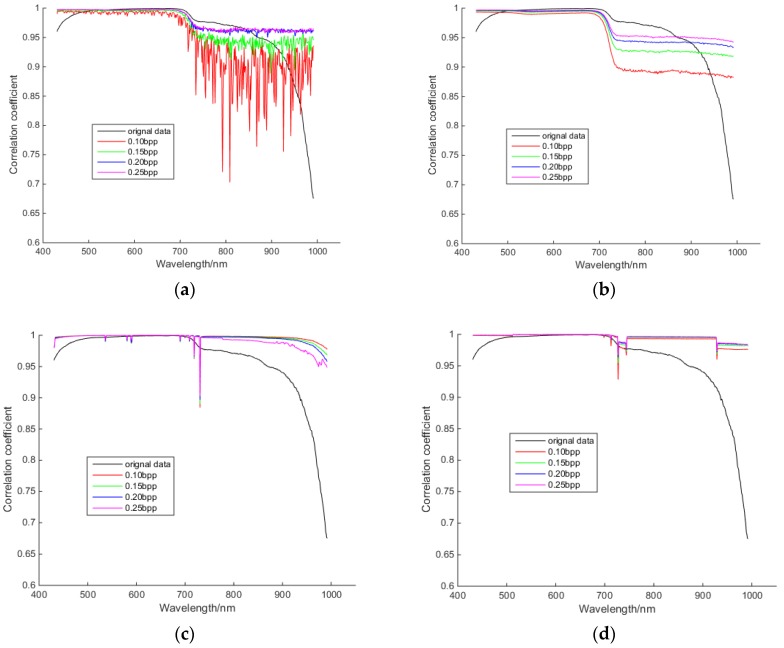
Interspectral correlation of reconstructed tea hyperspectral images for different algorithms at different compression rates. (**a**) SSCS; (**b**) BHCS; (**c**) AGDCS; (**d**) PSSAHCS.

Interspectral correlations of hyperspectral images are actually much higher than their spatial correlations. Figure 20 shows the spectral correlation curves of reconstructed tea hyperspectral images of different algorithms at different compression rates. It shows that the ends of the spectral correlation curves of original tea hyperspectral images decrease significantly. Additionally, the interspectral correlations of reconstructed tea hyperspectral images of PSSAHCS are better than those of SSCS, BHCS and AGDCS, especially for those bands with wavelengths larger than 700 nm.

## 4. Conclusions

Spatial adaptive blocking, which is based on the row and column correlations of hyperspectral images, can utilize the spatial correlation effectively. Spectral adaptive grouping divides the bands with high spectral correlation into the same group, so that it can make full use of interspectral correlation. Moreover, the prediction-based strategy is based on the linear model to denoise the hyperspectral images significantly. Therefore, the proposed PSSAHCS algorithm shows huge potential for hyperspectral images.

## Figures and Tables

**Table 1:** The average PSNR of reconstructed tea hyperspectral images at different bit rates.

Different Algorithms	Average PSNR of Reconstructed Tea Hyperspectral Images (dB)			
	Bit Rates			
	0.10 bpp	0.15 bpp	0.20 bpp	0.25 bpp
SSCS	31.0994	32.4488	33.3739	33.5721
BHCS	32.2594	32.8965	33.4452	33.6834
AGDCS	32.5154	32.8186	33.0399	33.3976
PSSAHCS	34.6838	34.9093	35.0225	35.0945
